# Associations between polymorphisms of the ADIPOQ gene and hypertension risk: a systematic and meta-analysis

**DOI:** 10.1038/srep41683

**Published:** 2017-02-09

**Authors:** Weina Fan, Xiaowei Qu, Jing Li, Xingning Wang, Yanping Bai, Qingmei Cao, Liqun Ma, Xiaoyao Zhou, Wei Zhu, Wei Liu, Qiang Ma

**Affiliations:** 1 Department of Cardiology, Centre Hospital of Xianyang, Xianyang 712000, People’s Republic of China; 2 Department of Clinical Laboratory, The Affiliated Hospital of Yan’an University, Yan’an University, Yan’an 71600, People’s Republic of China; 3 Department of Infection, Renmin Hospital of Yan’an, Yan’an 716000, People’s Republic of China; 4 Department of Cardiology, Affiliated Hospital of Yan’an University, Yan’an University, Yan’an 71600, People’s Republic of China; 5 Department of Nephropathy, 2^nd^ Affiliated Hospital of Xi’an Jiaotong University, Xi’an Jiaotong University, Xi’an 710004, People’s Republic of China; 6 Department of Invasive Technology, Traditional Chinese Medicine hospital of Shanxi, Xi’an 710003, People’s Republic of China; 7 Department of Clinical laboratory, Centre hospital of Baoji, Baoji 721008, People’s Republic of China; 8 Department of Vascular Disease and Hypertension, Peripheral Vascular, 1^st^ Affiliated Hospital of Xi’an Jiaotong University, Xi’an 710061, People’s Republic of China

## Abstract

ADIPOQ gene polymorphisms have been indicated to be associated with hypertension; however, published studies have reported inconsistent results. Eligible studies were retrieved by searching the PubMed, Embase and China National Knowledge Infrastructure databases. The case group consisted of patients with hypertension, and the control group consisted of subjects with normal blood pressure. Based on eleven published articles, involving 4837 cases and 5618 controls, the pooled results from rs2241766 polymorphism showed increased risk in the allelic model (G VS T: OR = 1.16, 95%CI = 1.06–1.27), recessive model (GG VS GT + TT: OR = 1.34, 95%CI = 1.10–1.63), dominant model (GG + GT VS TT: OR = 1.15, 95%CI = 1.02–1.30) and homozygote model (GG VS TT: OR = 1.38, 95%CI = 1.21–1.69). In addition, rs266729 polymorphism showed increased risk for hypertension in the recessive model (GG VS GC + CC: OR = 1.43, 95%CI = 1.02–2.01). In the Caucasian subgroup, rs1501299 polymorphism showed decreased risk of hypertension in the allelic model (T VS G: OR = 0.75, 95%CI = 0.58–0.97), dominant model (TT + TG VS GG: OR = 0.83, 95%CI = 0.71–0.98) and heterozygote model (TG VS GG: OR = 0.82, 95%CI = 0.68–0.99). The rs2241766 polymorphism was associated with a significant increase in hypertension risk based on our analysis. Moreover, an increased risk of rs266729 in hypertension patients was also detected. Our meta-analysis suggests that the rs1501299 polymorphism may play a protective role in hypertension in Caucasian subgroup; however, this finding requires further study.

Hypertension is a major risk factor for vascular disease and the leading source of global health burden^1^. Although the aetiology of hypertension has not been fully elucidated, interactions between genes and environmental factors are suggested to play an important role in the pathological process of hypertension^2^. In recent years, genes and their polymorphisms have been found to be associated with blood pressure and susceptibility of hypertension^3^,^4^,^5^,^6^.

Adiponectin is an adipocyte-derived hormone and plays an important role in energy homeostasis by regulating glucose and lipid metabolism^7^. Decreases in plasma adiponectin levels are observed in cardiovascular disease, essential hypertension, obesity and type II diabetes^8^,^9^,^10^, which may suggest a special role of adiponectin in the pathogenesis of hypertension. The ADIPOQ gene is located on chromosome 3q27 and encodes the protein adiponectin. Several single-nucleotide polymorphisms (SNPs) in the ADIPOQ gene have been described^11^. These polymorphisms in the ADIPOQ gene can modulate circulating concentration of adiponectin^12^,^13^,^14^.

Most of the published studies have investigated the associations of rs2241766, rs1501299 and rs266729 polymorphisms in the ADIPOQ gene with risk factors of hypertension^15^,^16^,^17^,^18^. However, the results are inconsistent. Wei *et al*.^17^ reported that the polymorphisms of 45 T/G (rs2241766) and 276 G/T (rs1501299) in the ADIPOQ gene were not associated with hypertension, but Mousavinasab *et al*.^19^ reported opposite findings. Moreover, polymorphism of 11377 C/G (rs266729) in ADIPOQ gene was indicated to have independent effects on diastolic blood pressure reported by Avery *et al*.^16^. The small numbers and varying populations of the published studies may partially account for the controversial results. Therefore, we performed this meta-analysis to investigate the associations between ADIPOQ rs2241766, rs1501299 and rs266729 polymorphisms and hypertension.

## Results

### The Characteristics of the Include Studies

A total of 233 articles were obtained by online and manual search. After removing duplicates and screening the title and abstract, eighteen articles were selected. Seven articles were excluded due to lack of detailed genotype distribution. Finally, a total of eleven published articles^10^,^17^,^18^,^20^,^21^,^22^,^23^,^24^,^25^,^26^,^27^, involving 4837 cases and 5618 controls were included in this meta-analysis (Seen in the Table S2 PRISMA Flow Diagram^28^).

The characteristics of all the included articles are summarized in Table 1. For rs2241766, 12 studies were included with 2147 cases and 2601 controls; 9 studies with 1859 cases and 2109 controls are included for rs1501299; only 5 studies with 831 cases and 908 controls concerning the rs266729 and hypertension risk are included.

### Meta-Analysis Results and Heterogeneity Analysis

**Figure 1:**
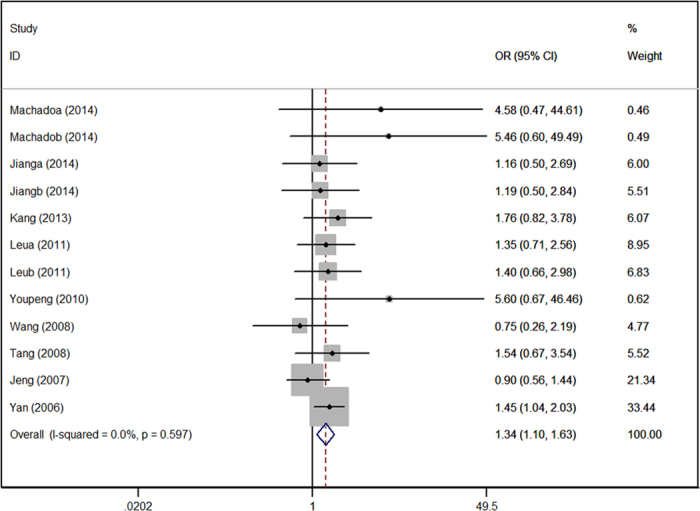
Forest plot of hypertension risk associated with the GG genotype in ADIPOQ rs2241766 polymorphism. OR = odd ration, CI = confidence interval.

**Figure 2:**
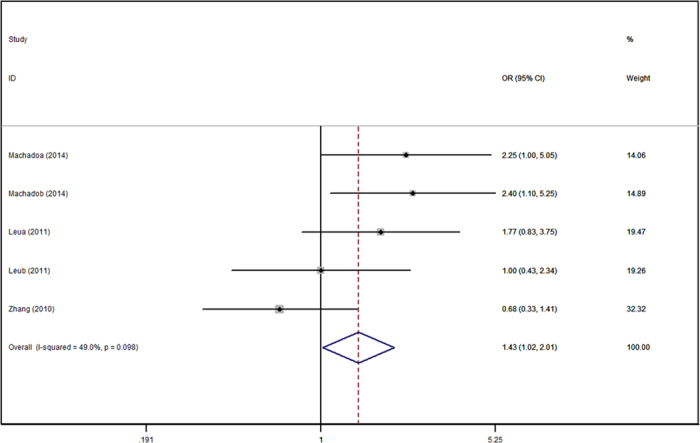
Forest plot of hypertension risk associated with the T allele compared with the G allele in ADIPOQ rs1501299 polymorphism. OR = odd ration, CI = confidence interval.

**Figure 3:**
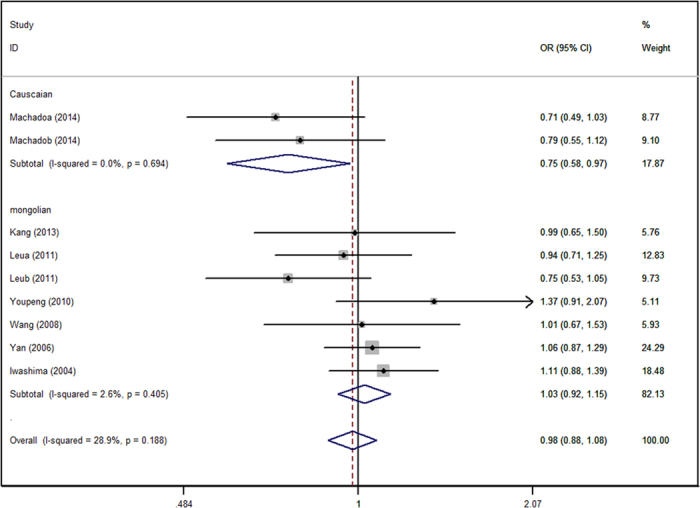
Forest plot of hypertension risk associated with the GG genotype in ADIPOQ rs266729 polymorphism in subgroup analysis of ethnicity. OR = odd ration, CI = confidence interval.

Table 2 shows the main results of this meta-analysis and the heterogeneity of the ADIPOQ gene polymorphisms and hypertension risk. The fixed effect model was adopted to calculate the pooled ORs for each individual polymorphism lacking heterogeneity. The rs2241766 polymorphism was associated with increased risk of hypertension in the allelic model (G VS T: OR = 1.16, 95%CI = 1.06–1.27), recessive model (GG VS GT + TT: OR = 1.34, 95%CI = 1.10–1.63) (Fig. 1), dominant model (GG + GT VS TT: OR = 1.15, 95%CI = 1.02–1.30), and homozygote model (GG VS TT: OR = 1.38, 95%CI = 1.21–1.69). For rs266729, increased risk was only found in the recessive model (GG VS GC + CC: OR = 1.43, 95%CI = 1.02–2.01) (Fig. 2). Association was not detected in rs1501299 polymorphism, and subsequently, subgroup analysis was introduced to uncover some potential details concerning associations between rs1501299 and hypertension risk. Table 3 summarizes the results of the stratified analyses of ADIPOQ polymorphisms and hypertension risk. As stratified by ethnicity for rs1501299, significant associations were found in the Caucasian group. A decreased risk of hypertension was observed in the allelic model (T VS G: OR = 0.75, 95%CI = 0.58–0.97) (Fig. 3), dominant model (TT + TG VS GG: OR = 0.83, 95%CI = 0.71–0.98), and heterozygote model (TG VS GG: OR = 0.82, 95%CI = 0.68–0.99). However, when stratified by age, body mass index, source of controls and sample size, no significant association was found. Significant results were also observed in both Caucasian and Mongolian subgroups in rs2241766 and rs266729 polymorphisms (Table 3).

### Sensitivity analysis

**Figure 4:**
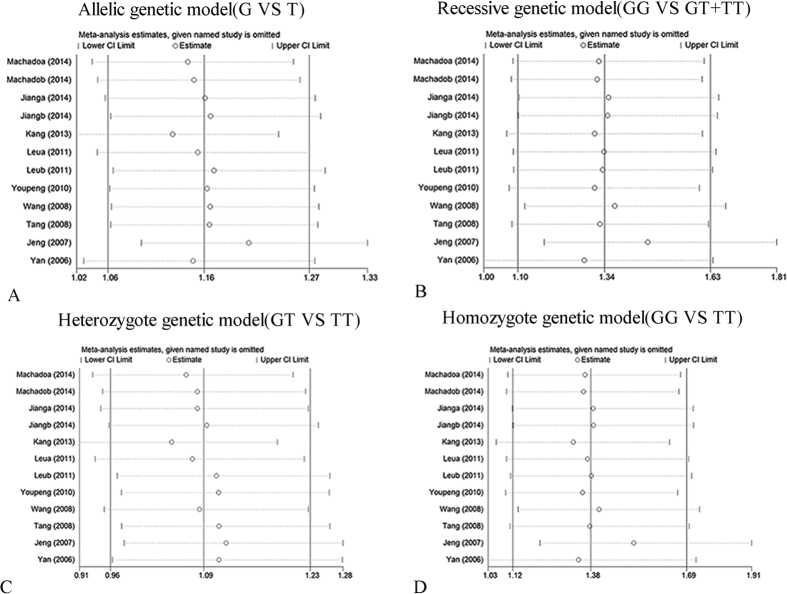
Influence analysis of rs2241766 polymorphism and hypertension risk. (**A**) Allelic genetic model analysis. (**B**) Recessive genetic model analysis. (**C**) Heterozygote genetic model analysis. (**D**) Homozygote genetic model analysis.

**Figure 5:**
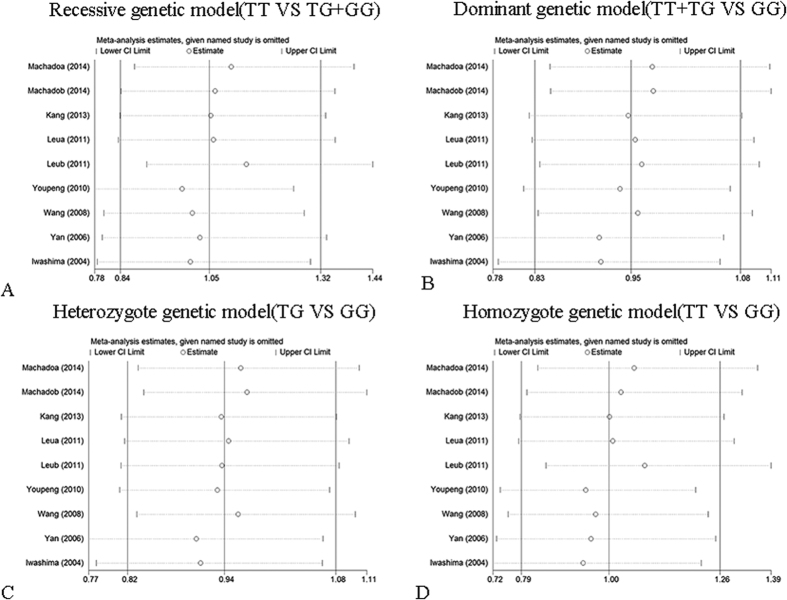
Influence analysis of rs1501299 polymorphism and hypertension risk. (**A**) Recessive genetic model analysis. (**B**) Dominant genetic model analysis. (**C**) Heterozygote genetic model analysis. (**D**) Homozygote genetic model analysis.

**Figure 6:**
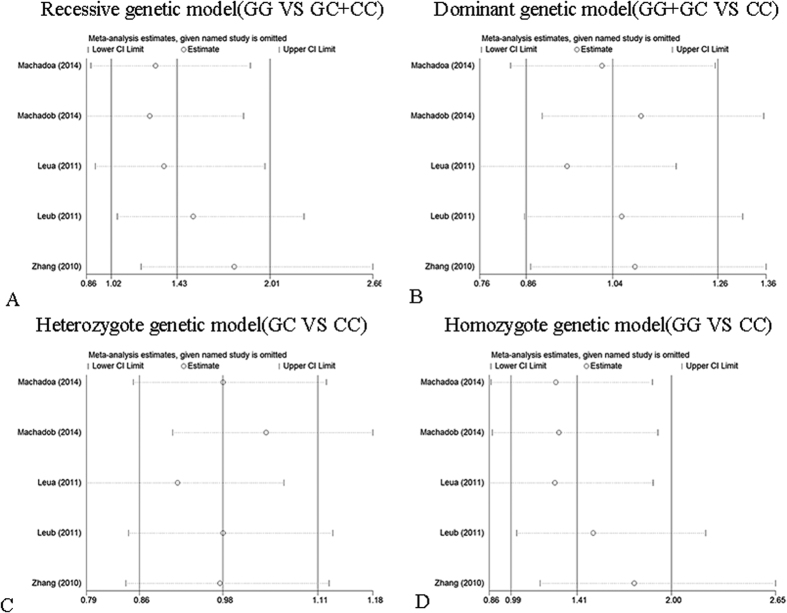
Influence analysis of rs266729 polymorphism and hypertension risk. (**A**) Recessive genetic model analysis. (**B**) Dominant genetic model analysis. (**C**) Heterozygote genetic model analysis. (**D**) Homozygote genetic model analysis.

Sensitivity analysis was performed by sequentially omitting 1 individual study; in order to detect the influence of each study on the overall meta-analysis. No heterogeneity was observed in the polymorphisms (Figs 4, 5 and 6), thus the results of our meta-analysis were stable.

### Publication bias

**Figure 7:**
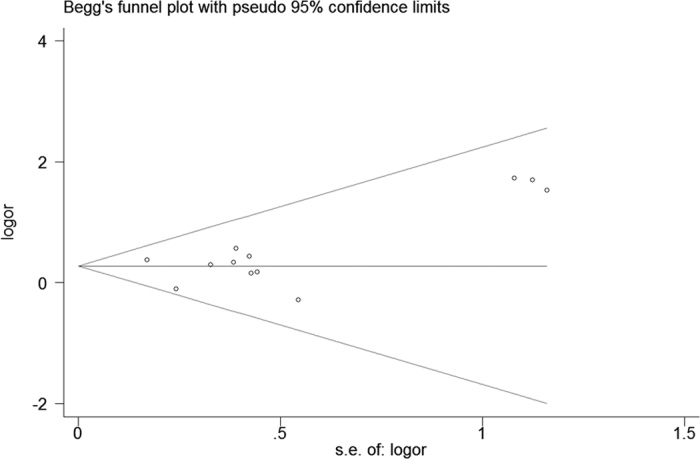
Begg’s funnel plot for contrast in overall analysis in recessive model. Each point represents a separate study for the indicated association. Size graph symbol by weighs. Log [OR] natural logarithm of OR. Horizontal line means effect size.

No publication bias was detected among studies regarding the association between the rs2241766 polymorphism and hypertension risk (Fig. 7). However, for rs1501299 and rs266729, publication bias was not evaluated because the number of studies included was less than 10^29^.

## Discussion

Endothelial dysfunction plays an important role in the pathogenesis of hypertension^30^,^31^,^32^,^33^. Adiponectin modulates the endothelial inflammatory response *in vitro*, and its concentration is decreased in patients with coronary artery disease and hypertension^34^,^35^. The low circulating level of adiponectin was found to be related to hypertension^36^,^37^, and polymorphisms of the ADPIOQ gene was reported to have a strong association with adiponectin concentration^38^. Whether the polymorphisms of the ADIPOQ gene are associated with hypertension have attracted growing increased attention. Although there are many researches about the SNPs in ADIPOQ with hypertension, three common gene variants of ADIPOQ, which are rs2241766 (+45 T > G in exon2), rs1501299 (+276 G > T in intron2) and rs266729 (−11377 C > G in proximal promoter region), are widely deeply studied and full of inconsistent results, besides, the data about the three SNPs is sufficient to conduct meta-analysis or subgroup-analysis. Therefore, we choose these three polymorphisms to explore the associations between ADIPOQ gene polymorphisms with hypertension risk in our meta-analysis.

Our results show that the rs2241766 polymorphism in the ADIPOQ gene is significantly associated with hypertension. Increased risk is observed in the allele, recessive, dominant and homozygote genetic model. The minor G allele of ADIPOQ rs2241766 may increase the risk of hypertension by 16% (P = 0.002, OR = 1.16, 95%CI = 1.06–1.27), and GG genotype may increase the risk of hypertension by 34% compared to the GT and TT genotype (P = 0.004, OR = 1.34, 95%CI = 1.10–1.63). When compared with the TT genotype alone, a 38% increase in risk was detected in GG genotype group (P = 0.003, OR = 1.38, 95%CI = 1.21–1.69). In the ethnicity subgroup analysis, increased risk is not only observed in the allele, recessive, dominant and homozygote genetic models in the Caucasian group but also in allele, recessive and homozygote genetic model in Mongolian population. In the eleven included studies, four studies from the Chinese population are consistent with our results^18^,^21^,^23^,^26^. An increase in hypertension risk attributed to rs2241766 polymorphism was found in the Chinese Bai population and a significant association with serum total triglyceride and low density lipoprotein was also detected in patients with high blood pressure reported by Kang *et al*.^26^. In addition, the GG + GT genotype was related to high hypertension susceptibility and low circulating adiponectin level when compared to the TT genotype reported by Tang *et al*.^23^. However, the association between rs2241766 polymorphism and plasma adiponectin level was found to be only significant in normal blood pressure group reported by Jeng *et al*.^18^. In his opinion, no association between hypertension and rs2241766 polymorphism may be attribute to environmental factors such as diet-induced obesity. The rs2241766 polymorphism at exon2 in ADIPOQ gene may have an influence on the mRNA level in adipose tissue^39^. It is postulated that the rs2241766 polymorphism may affect the expression of ADIPOQ gene and contribute to the low level of adiponectin in plasma.

Significant association between rs266729 and hypertension is found in the recessive genetic model in our study. The GG genotype may increase the risk of hypertension by 43% compared to GC and CC genotype (P = 0.040, OR = 1.43, 95%CI = 1.02–2.01). In the ethnicity subgroup analysis, increased risk was only detected in the recessive and homozygote genetic model. Four of five included studies addressing the association between rs266729 and hypertension risk were consistent with our result. Haplotype is also an important risk factor in hypertension and the −11426G −11377C haplotype was found to be associated with low plasma adiponectin level in the hypertension group, as reported by Zhang *et al*.^20^. The CG genotype of rs266729 was associated with high blood pressure in female group reported by Machado *et al*.^24^. In addition, a tendency of increased in DBP towards the CG and GG genotypes of the rs266729 polymorphism in healthy pregnancy women was also detected in his study. The G allele of rs266729 was significantly associated with the increased risk of hypertension by Jiang *et al*.^27^. Moreover, not only in Chinese group, an increase in SBP^16^ and DBP^15^ caused by rs266729 polymorphism was detected in a British population. Replacing a C with a G in the rs266729 polymorphism occurs in the proximal promoter region and genetic polymorphisms in the promoter region of ADIPOQ are associated with low serum adiponectin level and increased risk of hypertension in a Hong Kong Chinese population^15^. Based on the present evidence, rs266729 polymorphism is significantly associated with hypertension, however, the mechanism of whether the rs266729 polymorphism has an influence on hypertension susceptibility through the low levels of plasma adiponectin warrants further study.

In the Caucasian subgroup, the rs1501299 polymorphism in the ADIPOQ gene was significantly associated with hypertension. The minor T allele of ADIPOQ rs266729 may decrease the risk of hypertension by 25% (P = 0.026, OR = 0.75, 95%CI = 0.58–0.97), and the TG genotype may decrease the risk of hypertension by 18% compared to GG genotype (P = 0.112, OR = 0.82, 95%CI = 0.68–0.99). The same decreased risk of hypertension was also observed in the dominant genetic model. For the rs1501299 polymorphism, a protective role of rs1501299 polymorphism in hypertension group is found in the Caucasian subgroup, which is contrary to the results of nine included studies. An association between the rs1501299 polymorphism and adiponectin concentration was found in obese subjects (BMI ≥ 26.7 Kg/m^2^)^40^. High serum adiponectin in T-allele carriers of rs1501299 polymorphism in the severe preeclampic group was observed by Youpeng *et al*.^21^. However, in our study, no significant association was found in the BMI subgroup and gender could not be stratified by subgroup analysis due to lack of details regarding the gender distribution. Although the plasma adiponectin level is low in hypertension patients, we hypothesized that the ability of rs1501299 polymorphism to reduce adiponectin level is weaker than the other polymorphisms, thereby allowing for a relatively higher level of serum adiponectin, which suggests to a protective role for this polymorphism hypertension. More studies are required to explore the precise protective mechanism of rs1501299 polymorphism in hypertension.

There were several limitations in this meta-analysis. First, only English and Chinese articles were included, which may bias the results. Second, patient heterogeneity and confounding factors might have distorted the analysis. Third, the number of included studies was relatively small in some subgroups, and, the results should be interpreted with caution. Fourth, the deviation of genotype distributions from HWE in the control populations in two studies addressing the association between rs2241766 and hypertension may imply genotyping errors or control selection bias in those studies. Fifth, only three common SNPs were evaluated in our study and other relevant SNPs in ADIPOQ which are unknown or understudied or not studies at all may also have potential associations with hypertension. Sixth, most included studies were researches about the ADIPOQ polymorphisms with systolic blood pressure. Whether there are potential relationship between the ADIPOQ polymorphisms with specific hypertension and related outcomes (diastolic blood pressure and pulse pressure) requires additional study. In addition, the potential influence on genotype-hypertension associations by environment factors is worthy of consideration.

In conclusion, the rs2241766 polymorphism was found to be associated with a significant increase in hypertension risk based on our analysis. Moreover, an increased risk in rs266729 in hypertension patients was also detected. Our meta-analysis suggests that rs1501299 polymorphism may play a protective role in hypertension in the Caucasian subgroup; however, additional studies are required to support this finding.

## Methods

The systematic review was written in adherence to the PRISMA (Preferred Reporting Items for Systematic Reviews and Meta-analyses) checklist^41^. Ethical approval was not necessary according to local legislation because of the type of study (meta-analysis)^42^.

### Identification of the related Studies

PubMed, Embase, VIP, Wanfang and China National Knowledge Infrastructure databases were thoroughly searched in March 2016 by the first two investigators to identify potential studies addressing the associations between the ADPIOQ gene polymorphisms and hypertension. The terms “hypertension,” “high blood pressure,” “adiponectin,” “ADIPOQ,” “APM1,” “polymorphism,” and “polymorphisms” were used. The missing data (the data that we failed to identify during the electronic search) were obtained by reviewing the citations of review articles and all eligible studies.

### Inclusion and Exclusion criteria

To be included in this meta-analysis, studies met the following inclusion criteria: (1) evaluation of the association between the ADIPOQ gene polymorphisms and hypertension; (2) case-control study or cohort design; (3) detailed genotype data could be acquired to calculate odds ratios (ORs) and 95% confidence intervals (CIs); Exclusion criteria: (1) duplication of previous publications; (2) comment, review and editorial; (3) study without detailed genotype data. The selection of the studies was achieved by two investigators independently, according to the inclusion and exclusion criteria by screening the title, abstract and full-text. Any dispute was solved by discussion.

### Data Extraction

From each study, the following data were independently extracted by the first two investigators using a standardized form: first author’s last name, year of publication, study country, ethnicity, genotyping methods, age, body mass index, sample size, source of controls, Hardy-Weinberg equilibrium, number of cases and controls, and genotype frequency in cases and controls for ADIPOQ gene. Different ethnicity descents were classified as Caucasian and Mongolian. Disagreements were resolved through discussion.

### Quality assessment

Two reviewers independently assessed the quality of the included studies, according to a set of criteria (Table S1) modified on the basis of the Newcastle-Ottawa quality assessment scale. Scores ranged from 0 to 10, with 0 as the lowest and 10 as the highest quality.

### Statistics analysis

Hardy–Weinberg equilibrium (HWE) was evaluated for each study by Chi-square test in control groups, and P < 0.05 was considered as a significant departure from HWE. Odds ratio (OR) and 95% confidence intervals (CIs) were calculated to evaluate the strength of the association between ADIPOQ gene polymorphisms and hypertension risk. Pooled ORs were performed for the allelic model (rs2241766: G versus T; rs1501299: T versus G; rs266729: G versus C), recessive model (rs2241766: GG versus GT + TT; rs1501299: TT versus TG + GG; rs266729: GG versus GC + CC), dominant model (rs2241766: GG + GT versus TT; rs1501299: TT + TG versus GG; rs266729: GG + GC versus CC), heterozygote model (rs2241766: GT versus TT; rs1501299: TG versus GG; rs266729: GC versus CC), and homozygote model (rs2241766: GG versus TT; rs1501299: TT versus GG; rs266729: GG versus CC), respectively. Heterogeneity was evaluated by Q statistic (significance level of P < 0.1) and I^2^ statistic (greater than 50% as evidence of significant inconsistency). Heterogeneity between studies was evaluated with the I^2^ test, and a higher I^2^ values means higher levels of heterogeneity (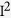 > 90%: extreme heterogeneity; 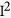 = 70% to 90%: large heterogeneity; 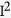
=50% to 70%: moderate heterogeneity; 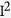 < 50%: no heterogeneity). In heterogeneity evaluation, when the 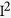 < 50%, the fixed-effects model would be used; if the 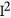
=50% to 90%, a random-effects model was used; if the 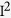 > 90%, the studies would not be pooled. Whenever heterogeneity was significant, sensitivity analysis was performed to detect the heterogeneity by omitting each study in each turn. Besides, subgroup analyses were stratified by ethnicity (Caucasian, Mongolian), age, body mass index, source of controls and sample size. The potential for publication bias was assessed with Begg’s funnel plot and Egg’s test. All the tests in this meta-analysis were conducted with the STATA software (version 12.0; State Corporation, College Station, Texas, USA). To adjust for multiple comparisons, we applied the Bonferroni method^43^, which control for the false discovery rate (FDR). The power of meta-analysis for each SNP to detect some effect size was estimated according to the method recommended by Hedges and Piggott, given a significant value of 0.05^44^.

## Additional Information

**How to cite this article:** Fan, W. *et al*. Associations between polymorphisms of the ADIPOQ gene and hypertension risk: a systematic and meta-analysis. *Sci. Rep.* **7**, 41683; doi: 10.1038/srep41683 (2017).

**Publisher's note:** Springer Nature remains neutral with regard to jurisdictional claims in published maps and institutional affiliations.

## Supplementary Material

Supplementary Table S1

Supplementary Table S2

Supplementary Table S3

## Figures and Tables

**Table 1:** Characteristics of included studies selected for meta-analysis.

Study	Year	Country	Ethnicity	Age, mean ± SD, year		Body mass index (kg/m^2^)		Type of study	Sample size		Control source	Genotype distributions in cases/controls			Genotyping method	Quality score	HWE^*^
				Case	Control	Case	Control		Case	Control		11	12	22			
**rs2241766**																	
Machado GH	2014	Brazil	Caucasian	26 ± 4.5	24 ± 4	27.53 ± 5.15	22.80 ± 2.7	Case-control	113	169	HB	81/139	29/29	3/1	PCR	8	0.697
Machado PE	2014	Brazil	Caucasian	27 ± 4.5	24 ± 5	26.28 ± 4.8	22.80 ± 2.7	Case-control	127	169	HB	97/139	26/29	4/1	PCR	8	0.697
Jiang HT	2014	China	Mongolian	68.8 ± 6.7	67.1 ± 7.1	24.5 ± 4.3	23.1 ± 3.3	Case-control	202	166	PB	102/91	86/65	14/10	PCR	10	0.720
Jiang HT + DM	2014	China	Mongolian	68.2 ± 6.1	67.1 ± 7.2	26.0 ± 3.7	23.1 ± 3.3	Case-control	169	166	PB	90/91	67/65	12/10	PCR	10	0.720
Kang	2013	China	Mongolian	NA	NA	22.57 ± 2.27	21.53 ± 2.17	Case-control	153	126	HB	63/75	68/40	22/11	PCR	8	0.105
Leu HT	2011	China	Mongolian	41.4 ± 0.7	46.7 ± 0.8	25.4 ± 0.3	23.5 ± 0.1	Family-based	159	446	PB	74/233	70/181	15/32	PCR	10	0.696
Leu HT + Mets	2011	China	Mongolian	41.7 ± 0.6	57.5 ± 1.0	28.3 ± 0.3	27.1 ± 0.3	Family-based	192	165	PB	92/78	81/75	19/12	PCR	9	0.696
Youpeng	2010	China	Mongolian	30.9 ± 6.0	30.0 ± 4.5	22.05 ± 2.65	20.1 ± 1.8	Case-control	107	81	HB	74/53	26/27	7/1	PCR	7	0.228
Wang	2008	China	Mongolian	46.4 ± 14.0	46.8 ± 15.4	26.82 ± 2.44	22.43 ± 3.99	Case-control	80	160	HB	41/87	34/60	5/13	PCR	8	0.561
Tang	2008	China	Mongolian	63.5 ± 6.5	60.5 ± 7.1	NA	NA	Case-control	151	100	HB	99/63	32/28	20/9	PCR	8	0.036
Jeng	2007	China	Mongolian	51.6 ± 14.7	50.7 ± 12.1	25.7 ± 3.6	23.8 ± 3.4	Case-control	212	356	HB	82/126	99/173	31/57	PCR	9	0.853
Yan	2006	China	Mongolian	49 ± 9	48 ± 10	30.8 ± 2.5	22.4 ± 1.8	Case-control	482	497	HB	201/222	186/203	95/72	PCR	9	0.024
**rs1501299**																	
Machado GH	2014	Brazil	Caucasian	26 ± 4.5	24 ± 4	27.53 ± 5.15	22.80 ± 2.7	Case-control	113	161	HB	59/68	46/74	8/19	PCR	8	0.868
Machado PE	2014	Brazil	Caucasian	27 ± 4.5	24 ± 5	26.28 ± 4.8	22.80 ± 2.7	Case-control	127	161	HB	66/68	47/74	14/19	PCR	8	0.868
Kang	2013	China	Mongolian	NA	NA	22.57 ± 2.27	21.53 ± 2.17	Case-control	153	126	HB	100/82	46/38	7/6	PCR	8	0.560
Leu HT	2011	China	Mongolian	41.4 ± 0.7	46.7 ± 0.8	25.4 ± 0.3	23.5 ± 0.1	Family-based	159	446	PB	85/228	60/178	14/40	PCR	9	0.536
Leu HT + Mets	2011	China	Mongolian	41.7 ± 0.6	57.5 ± 1.0	28.3 ± 0.3	27.1 ± 0.3	Family-based	192	165	PB	117/93	68/56	7/16	PCR	9	0.089
Youpeng	2010	China	Mongolian	30.9 ± 6.0	30.0 ± 4.5	22.05 ± 2.65	20.1 ± 1.8	Case-control	107	81	HB	19/19	60/49	28/13	PCR	7	0.051
Wang	2008	China	Mongolian	46.4 ± 14.0	46.8 ± 15.4	26.82 ± 2.44	22.43 ± 3.99	Case-control	80	160	HB	41/74	28/73	11/13	PCR	8	0.392
Yan	2006	China	Mongolian	49 ± 9	48 ± 10	30.8 ± 2.5	22.4 ± 1.8	Case-control	482	497	HB	259/274	184/187	39/36	PCR	9	0.599
Iwashima	2004	Japan	Mongolian	59.4 ± 0.5	57.1 ± 0.6	24.4 ± 0.1	23.1 ± 0.2	Case-control	446	312	HB	225/165	180/124	41/23	PCR	9	0.964
**rs266729**																	
Machado GH	2014	Brazil	Caucasian	26 ± 4.5	24 ± 4	27.53 ± 5.15	22.80 ± 2.7	Case-control	113	161	HB	61/93	36/57	16/11	PCR	8	0.577
Machado PE	2014	Brazil	Caucasian	27 ± 4.5	24 ± 5	26.28 ± 4.8	22.80 ± 2.7	Case-control	127	161	HB	81/93	27/57	19/11	PCR	8	0.577
Leu HT	2011	China	Mongolian	41.4 ± 0.7	46.7 ± 0.8	25.4 ± 0.3	23.5 ± 0.1	Family-based	198	159	PB	85/84	90/64	23/11	PCR	9	0.800
Leu HT + Mets	2011	China	Mongolian	41.7 ± 0.6	57.5 ± 1.0	28.3 ± 0.3	27.1 ± 0.3	Family-based	173	159	PB	93/84	68/64	12/11	PCR	9	0.800
Zhang	2010	China	Mongolian	52.39 ± 9.60	49.25 ± 10.84	NA	NA	Case-control	220	268	HB	138/163	70/84	12/21	PCR	8	0.035

For rs2241766 variant, 11, 12 and 22 represent TT, GT, GG, respectively; for rs1501299 variant, 11, 12 and 22 represent GG, TG and TT, respectively; for rs266729 variant, 11, 12 and 22 represent CC, CG, and GG.

GH = gestational hypertension; PE = pre-eclampsia; HT = hypertension; DM = type 2 diabetes; Mets = metabolic syndrome; HB = hospital based; PB = population based; NA = not available.

*P value for Hardy–Weinberg equilibrium test in controls.

**Table 2:** Pooled ORs and 95%CIs of the associations between three polymorphisms in ADIPOQ gene and hypertension risk.

Genetic model	NO. of studies	OR	95%CI	Pmeta-analysis	Bon	FDR	Statistical method	I2 (%)	Paheterogeneity
rs2241766									
G VS T	12	1.16	1.06–1.27	0.002	0.010	0.007	Fixed	22.90	0.218
GG VS GT + TT	12	1.34	1.10–1.63	0.004	0.020	0.007	Fixed	0.00	0.597
GG + GT VS TT	12	1.15	1.02–1.30	0.024	0.120	0.030	Fixed	24.20	0.206
GT VS TT	12	1.09	0.96–1.23	0.204	1.000	0.204	Fixed	25.00	0.196
GG VS TT	12	1.38	1.12–1.69	0.003	0.015	0.007	Fixed	0.00	0.457
rs1501299									
T VS G	9	0.98	0.88–1.08	0.646	1.000	0.838	Fixed	28.90	0.188
TT VS TG + GG	9	1.05	0.84–1.32	0.670	1.000	0.838	Fixed	33.50	0.15
TT + TG VS GG	9	0.95	0.83–1.08	0.404	1.000	0.838	Fixed	0.00	0.458
TG VS GG	9	0.94	0.82–1.08	0.363	1.000	0.838	Fixed	0.00	0.647
TT VS GG	9	1.00	0.79–1.26	0.988	1.000	0.988	Fixed	37.50	0.119
rs266729									
G VS C	5	1.10	0.94–1.28	0.219	1.000	0.365	Fixed	29.70	0.224
GG VS GC + CC	5	1.43	1.02–2.01	0.040	0.200	0.140	Fixed	49.00	0.098
GG + GC VS CC	5	1.04	0.86–1.26	0.704	1.000	0.732	Fixed	19.70	0.289
GC VS CC	5	0.96	0.79–1.18	0.732	1.000	0.732	Fixed	41.90	0.142
GG VS CC	5	1.41	0.99–2.00	0.056	0.280	0.140	Fixed	44.20	0.127

OR = odd ratio; CI = confidence interval.

aP value for between-study heterogeneity based on Q test; Bon = p value in Bonferroni test; FDR = false discovery rate.

Significant results are marked in bold.

**Table 3:** Stratified analysis of the associations of ADIPOQ polymorphsims with hypertension.

Subgroup analysis	N	T VS G		TT VS TG + GG		TT + TG VS GG		TG VS GG		TT VS GG	
		OR (95%CI)	P_h_ (I^2^)	OR (95%CI)	P_h_ (I^2^)	OR (95%CI)	P_h_ (I^2^)	OR (95%CI)	P_h_ (I^2^)	OR (95%CI)	P_h_ (I^2^)
**rs1501299**											
**Ethnicity**											
Caucasians	2	0.75 (0.58,0.97)	0.694 (0.0%)	0.77 (0.47,1.27)	0.396 (0.0%)	0.83 (0.71,0.98)	0.975 (0.0%)	0.82 (0.68,0.99)	0.789 (0.0%)	0.68 (0.42,1.10)	0.445 (0.0%)
Mongolian	7	1.03 (0.92,1.15)	0.405 (2.6%)	1.05 (0.85,1.28)	0.157 (35.5%)	1.00 (0.93,1.08)	0.807 (0.0%)	1.00 (0.92,1.08)	0.891 (0.0%)	1.08 (0.87,1.34)	0.207 (29.0%)
**Age**											
≥40	5	1.00 (0.89,1.13)	0.398 (1.5%)	1.06 (0.80,1.39)	0.105 (47.7%)	0.99 (0.85,1.15)	0.705 (0.0%)	0.98 (0.84,1.15)	0.729 (0.0%)	1.04 (0.79,1.38)	0.137 (42.6%)
<40	3	0.89 (0.71,1.10)	0.044 (67.9%)	1.05 (0.68,1.61)	0.114 (54.0%)	0.77 (0.57,1.04)	0.179 (41.8%)	0.76 (0.55,1.05)	0.369 (0.0%)	0.89 (0.55,1.44)	0.064 (63.7%)
**BMI of cases** (**kg/m2)**											
≥26	5	0.91 (0.79,1.04)	0.190 (34.8%)	0.91 (0.67,1.23)	0.074 (53.2%)	0.88 (0.74,1.04)	0.324 (14.2%)	0.88 (0.74,1.06)	0.360 (8.1%)	0.84 (0.62,1.16)	0.082 (51.7%)
<26	4	1.07 (0.92,1.25)	0.482 (0.0%)	1.25 (0.90,1.75)	0.595 (0.0%)	1.04 (0.86,1.27)	0.696 (0.0%)	1.01 (0.82,1.25)	0.874 (0.0%)	1.24 (0.87,1.77)	0.508 (0.0%)
**Source of controls**											
Hospital based	7	1.01 (0.90,1.13)	0.205 (29.3%)	1.17 (0.91,1.50)	0.445 (0.0%)	0.97 (0.84,1.12)	0.299 (17.1%)	0.94 (0.81,1.10)	0.429 (0.0%)	1.11 (0.85,1.45)	0.308 (16.0%)
Population based	2	0.86 (0.69,1.07)	0.313 (1.8%)	0.69 (0.41,1.16)	0.072 (69.1%)	0.87 (0.66,1.15)	0.738 (0.0%)	0.93 (0.69,1.24)	0.828 (0.0%)	0.66 (0.39,1.14)	0.087 (65.8%)
**Sample size**											
Large (≥300)	4	1.00 (0.89,1.14)	0.255 (26.1%)	1.00 (0.75,1.33)	0.110 (50.4%)	1.01 (0.86,1.18)	0.650 (0.0%)	1.01 (0.86,1.19)	0.914 (0.0%)	1.00 (0.67,1.48)	0.160 (39.1%)
Small (<300)	5	0.93 (0.78,1.10)	0.156 (39.8%)	1.14 (0.79,1.64)	0.224 (29.6%)	0.82 (0.65,1.04)	0.387 (3.5%)	0.80 (0.62,1.02)	0.561 (0.0%)	1.00 (0.74,1.34)	0.101 (51.8%)
**rs2241766**		**G VS T**		**GG VS GT + TT**		**GG + GT VS TT**		**GT VS TT**		**GG VS TT**	
**Ethnicity**		**OR** (**95%CI)**	**P**_**h**_ (**I**^**2**^)	**OR** (**95%CI)**	**P**_**h**_ (**I**^**2**^)	**OR** (**95%CI)**	**P**_**h**_ (**I**^**2**^)	**OR** (**95%CI)**	**P**_**h**_ (**I**^**2**^)	**OR** (**95%CI)**	**P**_**h**_ (**I**^**2**^)
Caucasians	2	1.67 (1.16,2.40)	0.648 (0.0%)	4.92 (1.03,23.54)	0.915 (0.0%)	1.46 (1.07,2.00)	0.571 (0.0%)	1.37 (0.99,1.90)	0.511 (0.0%)	5.29 (1.11,25.28)	0.948 (0.0%)
Mongolian	10	1.13 (1.03,1.24)	0.353 (9.7%)	1.26 (1.06,1.50)	0.697 (0.0%)	1.05 (0.99,1.12)	0.281 (17.6%)	1.03 (0.96,1.10)	0.244 (21.7%)	1.25 (1.06,1.48)	0.527 (0.0%)
**rs266729**		**G VS C**		**GG VS GC + CC**		**GG + GC VS CC**		**GC VS CC**		**GG VS CC**	
**Ethnicity**		**OR** (**95%CI)**	**P**_**h**_ (**I**^**2**^)	**OR** (**95%CI)**	**P**_**h**_ (**I**^**2**^)	**OR** (**95%CI)**	**P**_**h**_ (**I**^**2**^)	**OR** (**95%CI)**	**P**_**h**_ (**I**^**2**^)	**OR** (**95%CI)**	**P**_**h**_ (**I**^**2**^)
Caucasians	2	1.13 (0.93,1.38)	0.423 (0.0%)	2.13 (1.28,3.54)	0.915 (0.0%)	0.97 (0.80,1.18)	0.238 (28.3%)	0.81 (0.63,1.04)	0.125 (57.4%)	1.87 (1.14,3.07)	0.859 (0.0%)
Mongolian	3	1.05 (0.91,1.20)	0.109 (54.8%)	1.06 (0.71,1.59)	0.204 (37.1%)	1.04 (0.92,1.19)	0.230 (31.9%)	1.05 (0.91,1.21)	0.451 (0.0%)	1.10 (0.74,1.63)	0.129 (51.1%)

OR = odd ratio; CI = confidence interval; BMI = Body mass index; P_h_ = P value for heterogeneity test; N = the number of studies.
