# Supplementary material for: Associations between polymorphisms of the ADIPOQ gene and hypertension risk: a systematic and meta-analysis
Source: Sci Rep. 2017 Feb 9;7:41683. doi: 10.1038/srep41683 (PMC5299502; doi:10.1038/srep41683)
Supplement: Supplementary Table S1 [file srep41683-s1.doc]

# Associations between polymorphisms of the ADIPOQ gene and hypertension risk: a systematic and meta-analysis

Authors

Weina Fan^1+^, M.D., Xiaowei Qu^2+^, B.D., Jing Li^3^, B.D., Xingning Wang^2^, B.D., Yanping Bai^4^, M.D., Qingmei Cao^2^, B.D., Liqun Ma^5^, B.D., Xiaoyao Zhou^6^, B.D., Wei Zhu^7&^, B.D., Wei Liu^4&^, B.D., Qiang Ma^8*^, PhD.

1. Department of Cardiology, Centre Hospital of Xianyang, Xianyang 712000, People’s Republic of China
2. Department of Clinical Laboratory, The Affiliated Hospital of Yan’an University, Yan’an University, Yan’an 71600, People’s Republic of China
3. Department of Infection, Renmin Hospital of Yan’an, Yan’an 716000, People’s Republic of China
4. Department of Cardiology, Affiliated Hospital of Yan’an University, Yan’an University, Yan’an 71600, People’s Republic of China
5. Department of Nephropathy, 2^nd^ Affiliated Hospital of Xi’an Jiaotong University, Xi’an Jiaotong University, Xi’an 710004, People’s Republic of China
6. Department of Invasive Technology, Traditional Chinese Medicine hospital of Shanxi, Xi’an 710003, People’s Republic of China
7. Department of Clinical laboratory, Centre hospital of Baoji, Baoji 721008, People’s Republic of China
8. Department of Vascular Disease and Hypertension, Peripheral Vascular, 1^st^ Affiliated Hospital of Xi’an Jiaotong University, Xi’an 710061, People’s Republic of China

*Corresponding author.

Tel.: +8613720771361(Q. Ma). E-mail address: maqiang197909@163.com

&Co-corresponding author.

Tel.: +8613891150019(W. Liu). E-mail address: liuwei1968@126.com

Tel.: +8613709178560(W. Zhu). E-mail address: zhuwei197112@126.com

| **Table S1.** **Scale for methodological quality assessment.** | |
| --- | --- |
| Criteria | Score |
| 1.Representativeness of cases |  |
| Hypertension diagnosed according to acknowledged criteria. | 2 |
| Mentioned the diagnosed criteria but not specifically described. | 1 |
| Not Mentioned. | 0 |
| 2.Source of controls |  |
| Population or community based | 3 |
| Hospital-based Hypertension-free controls | 2 |
| Healthy volunteers without total description | 1 |
| Hypertension-free controls with related diseases | 0.5 |
| Not described | 0 |
| 3.Sample size |  |
| >300 | 2 |
| 200-300 | 1 |
| <200 | 0 |
| 4.Quality control of genotyping methods |  |
| Repetition of partial/total tested samples with a different method | 2 |
| Repetition of partial/total tested samples with the same method | 1 |
| Not described | 0 |
| 5.Hardy-Weinberg equilibrium (HWE) |  |
| Hardy-Weinberg equilibrium in control subjects | 1 |
| Hardy-Weinberg disequilibrium in control subjects | 0 |
