# Supplementary material for: Associations between polymorphisms of the ADIPOQ gene and hypertension risk: a systematic and meta-analysis
Source: Sci Rep. 2017 Feb 9;7:41683. doi: 10.1038/srep41683 (PMC5299502; doi:10.1038/srep41683)
Supplement: Supplementary Table S2 [file srep41683-s2.doc]

# Associations between polymorphisms of the ADIPOQ gene and hypertension risk: a systematic and meta-analysis

Authors

Weina Fan^1+^, M.D., Xiaowei Qu^2+^, B.D., Jing Li^3^, B.D., Xingning Wang^2^, B.D., Yanping Bai^4^, M.D., Qingmei Cao^2^, B.D., Liqun Ma^5^, B.D., Xiaoyao Zhou^6^, B.D., Wei Zhu^7&^, B.D., Wei Liu^4&^, B.D., Qiang Ma^8*^, PhD.

1. Department of Cardiology, Centre Hospital of Xianyang, Xianyang 712000, People’s Republic of China
2. Department of Clinical Laboratory, The Affiliated Hospital of Yan’an University, Yan’an University, Yan’an 71600, People’s Republic of China
3. Department of Infection, Renmin Hospital of Yan’an, Yan’an 716000, People’s Republic of China
4. Department of Cardiology, Affiliated Hospital of Yan’an University, Yan’an University, Yan’an 71600, People’s Republic of China
5. Department of Nephropathy, 2^nd^ Affiliated Hospital of Xi’an Jiaotong University, Xi’an Jiaotong University, Xi’an 710004, People’s Republic of China
6. Department of Invasive Technology, Traditional Chinese Medicine hospital of Shanxi, Xi’an 710003, People’s Republic of China
7. Department of Clinical laboratory, Centre hospital of Baoji, Baoji 721008, People’s Republic of China
8. Department of Vascular Disease and Hypertension, Peripheral Vascular, 1^st^ Affiliated Hospital of Xi’an Jiaotong University, Xi’an 710061, People’s Republic of China

*Corresponding author.

Tel.: +8613720771361(Q. Ma). E-mail address: maqiang197909@163.com

&Co-corresponding author.

Tel.: +8613891150019(W. Liu). E-mail address: liuwei1968@126.com

Tel.: +8613709178560(W. Zhu). E-mail address: zhuwei197112@126.com


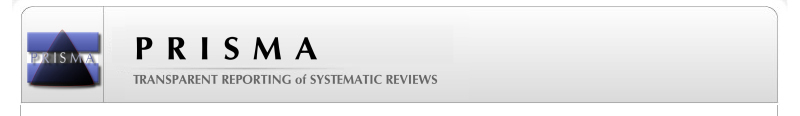
**PRISMA 2009 Flow Diagram**

**Screening**

**Included**

**Eligibility**

**Identification**

Records identified through PubMed and Embase database searching
(n = 202)

Additional records identified through, VIP, Wangfang, and CNKI database searching
(n = 31)

Records after duplicates removed
(n = 82)

Title and abstract screened
(n = 23)

Title and abstract excluded
(n = 59)

Full-text articles assessed for eligibility
(n = 18)

Full-text articles excluded,

Reviews, editorial

(n = 5)

Studies included in qualitative synthesis
(n = 11)

Studies included in quantitative synthesis (meta-analysis)
(n = 11)

Full-text articles excluded,

Lack of detailed genotype distribution data
(n = 7)
